# Stability and reactivity of Fe-DCA 2D metal–organic frameworks on chemically inert graphene support

**DOI:** 10.1039/d6ra03820d

**Published:** 2026-09-24

**Authors:** Dominik Hrůza, Jakub Planer, Tadeáš Lesovský, Ayesha Jabeen, Jan Čechal, Zdeněk Jakub

**Affiliations:** a CEITEC – Central European Institute of Technology, Brno University of Technology Purkyňova 123 612 00 Brno Czech Republic cechal@fme.vutbr.cz zdenek.jakub@ceitec.vutbr.cz; b Institute of Physical Engineering, Brno University of Technology Technická 2896/2 616 69 Brno Czech Republic

## Abstract

Atomically defined 2D Metal–Organic Frameworks (MOFs) show high promise for many applications, but their implementation is often hindered by their limited stability. Here, we provide an atomic-scale view on the structural stability and chemical reactivity of Fe-DCA 2D MOF placed on an inert graphene support. We use Scanning Tunneling Microscopy, Low-Energy Electron Microscopy/Diffraction, X-ray Photoemission Spectroscopy, and Density Functional Theory to unravel how the Fe-DCA reacts with oxygen and carbon monoxide in ultrahigh vacuum. We show that O_2_ exposure at room temperature causes structural collapse of the Fe-DCA framework, leaving oxidized Fe clusters and self-assembled DCA molecules on the surface. In contrast, CO does not stably adsorb on the defect-free Fe-DCA at room temperature, but larger CO doses induce slow disintegration of the Fe-DCA structure starting from domain boundaries and defects. Variable-temperature Scanning Tunneling Microscopy captures the atomic-scale details of this process at temperatures below 200 K, where the individual adsorbed CO molecules can be imaged. Overall, our results outline the stability limits of Fe-DCA networks on a technologically relevant graphene support; this knowledge is necessary for any potential application of similar materials, *e.g.*, in spintronics, catalysis, or sensing.

## Abstract 2

Scanning tunneling microscopy, X-ray photoemission spectroscopy, and DFT reveal how gas exposure triggers structural degradation of Fe-DCA on graphene, defining the intrinsic stability limits of this 2D MOF.

## Graphical abstract

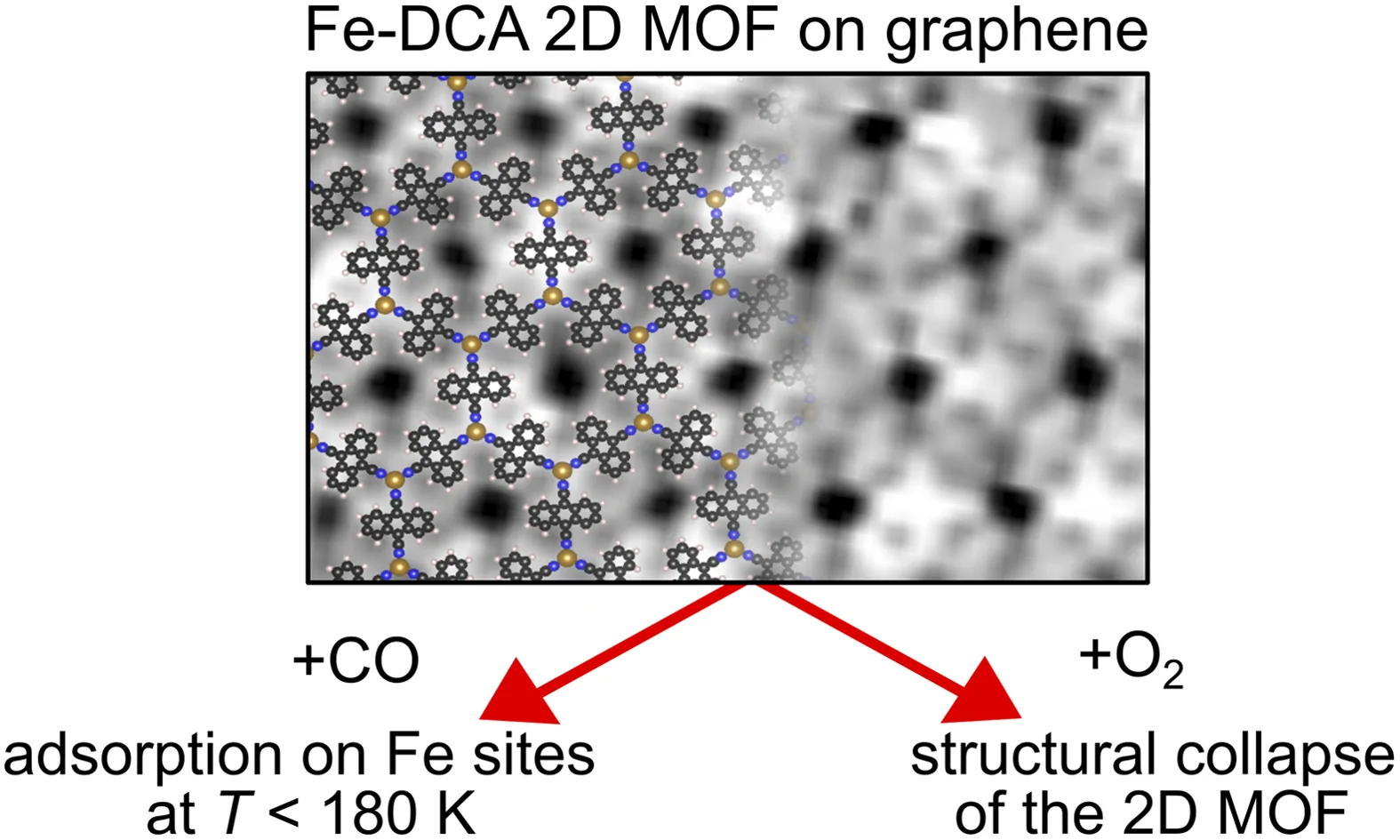

## Introduction

Metal–organic frameworks (MOFs) are tunable materials with promising applications in many fields, ranging from catalysis^1,2^ and sensing^2,3^ to electronics^2,4^ or spintronics.^2,5,6^ One way to study these materials with atomic-scale precision is to synthesize metal–organic monolayers on atomically flat surfaces, where they can be approached by scanning probe microscopy/spectroscopy techniques.^7,8^ Such an approach allows investigation of how the electronic structure, magnetic properties, and chemical reactivity depend on the structural details of the 2D MOF lattices,^9–15^ paving the way for tunable materials whose functionality stems from perfect atomic-scale control.

**Fig. 1:**
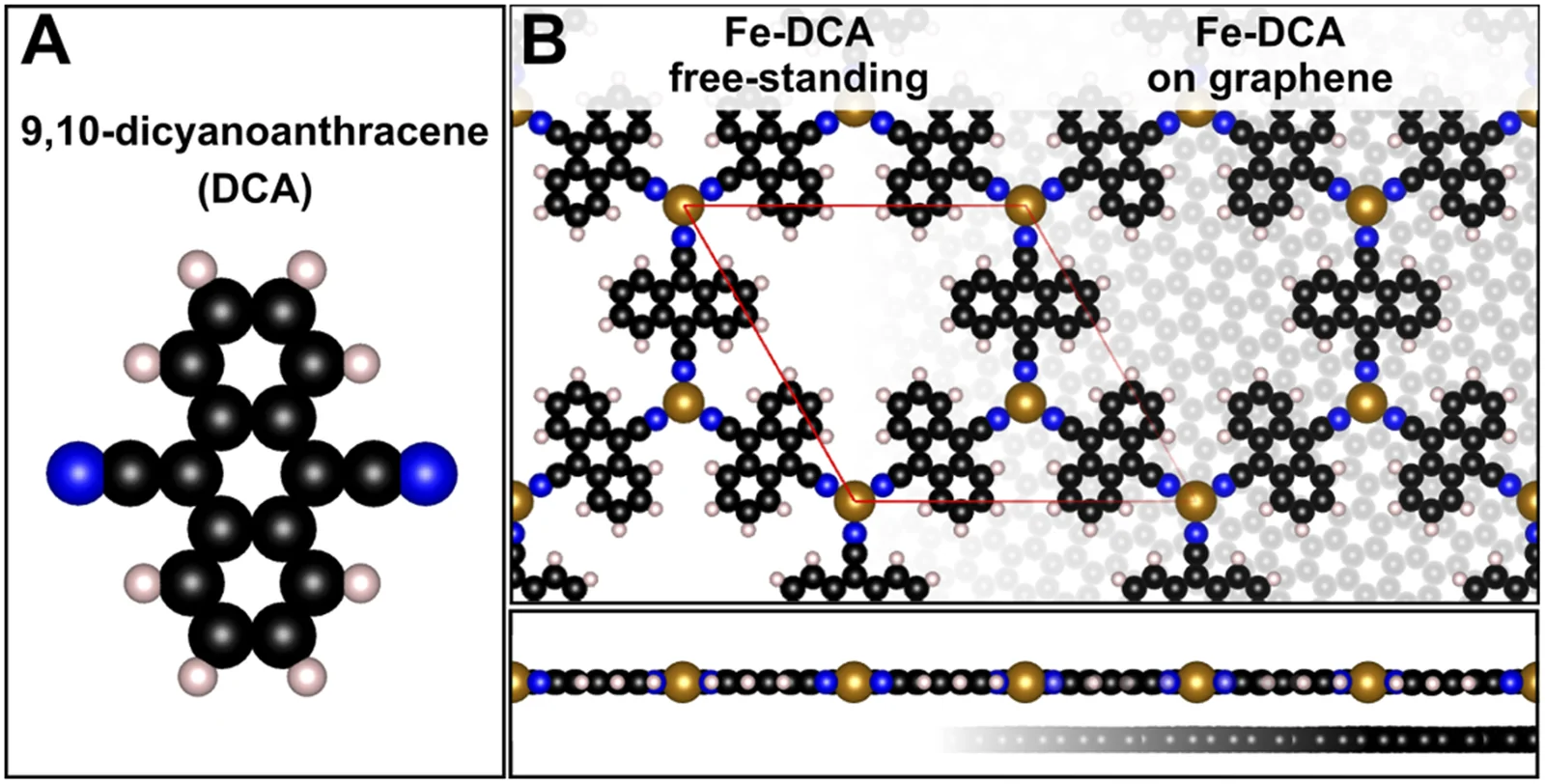
Structural models of (A) DCA linker molecule and (B) Fe-DCA 2D MOF in an idealized free-standing model (left) and supported on graphene (right). The DFT-optimized models reveal that the presence of graphene support does not significantly affect the Fe-DCA structure; in stark contrast to similar 2D MOFs supported on metals.^14,20–22^

Here, we provide an atomic-scale view on the stability and chemical reactivity of Fe-DCA 2D MOF (DCA = 9,10-dicyanoanthracene, see Fig. 1A). The Fe-DCA is relevant to both spintronics and catalysis-oriented research, as it has recently been demonstrated to be a 2D ferromagnet,^14^ and its reported structure features Fe cations coordinated to three nitrogen atoms, similar to the metal-N_3_ sites found in many single-atom catalysts.^16–19^ Our work aims to assess the intrinsic reactivity of the Fe-DCA and of the embedded Fe–N_3_ sites; thus, we synthesized it on a chemically inert graphene support. Such an approach minimizes the structural distortions that are typically present within 2D MOFs synthesized on metal surfaces.^14,20–22^ The lack of structural distortions is clearly seen in DFT-optimized models of free-standing and graphene-supported Fe-DCA shown in Fig. 1B.

To probe chemical reactivity of the Fe-DCA, we exposed it to molecular oxygen and carbon monoxide in ultrahigh vacuum. We monitored its evolution by Scanning Tunneling Microscopy (STM), X-ray Photoemission Spectroscopy (XPS) and Low-energy Electron Microscopy (LEEM), and we complemented the experimental results by Density Functional Theory (DFT) computations. We find that the Fe–N_3_ sites are highly reactive to O_2_, and the strong Fe–O_2_ interaction leads to structural collapse of the 2D MOF. In contrast, CO does not stably adsorb on the Fe–N_3_ sites within the Fe-DCA islands at room temperature, but it induces structural changes at the Fe-DCA island edges, leading to slow disintegration of the 2D MOF structure at high CO doses. Low-temperature STM experiments reveal that CO molecules can be stabilized at the Fe–N_3_ sites at temperatures below 200 K. Overall, our work addresses the stability limits of Fe-DCA on graphene, which is essential for potential applications of DCA-based 2D MOFs.

## Results and discussion

### Synthesis of Fe-DCA on Gr/Ir(111)

**Fig. 2:**
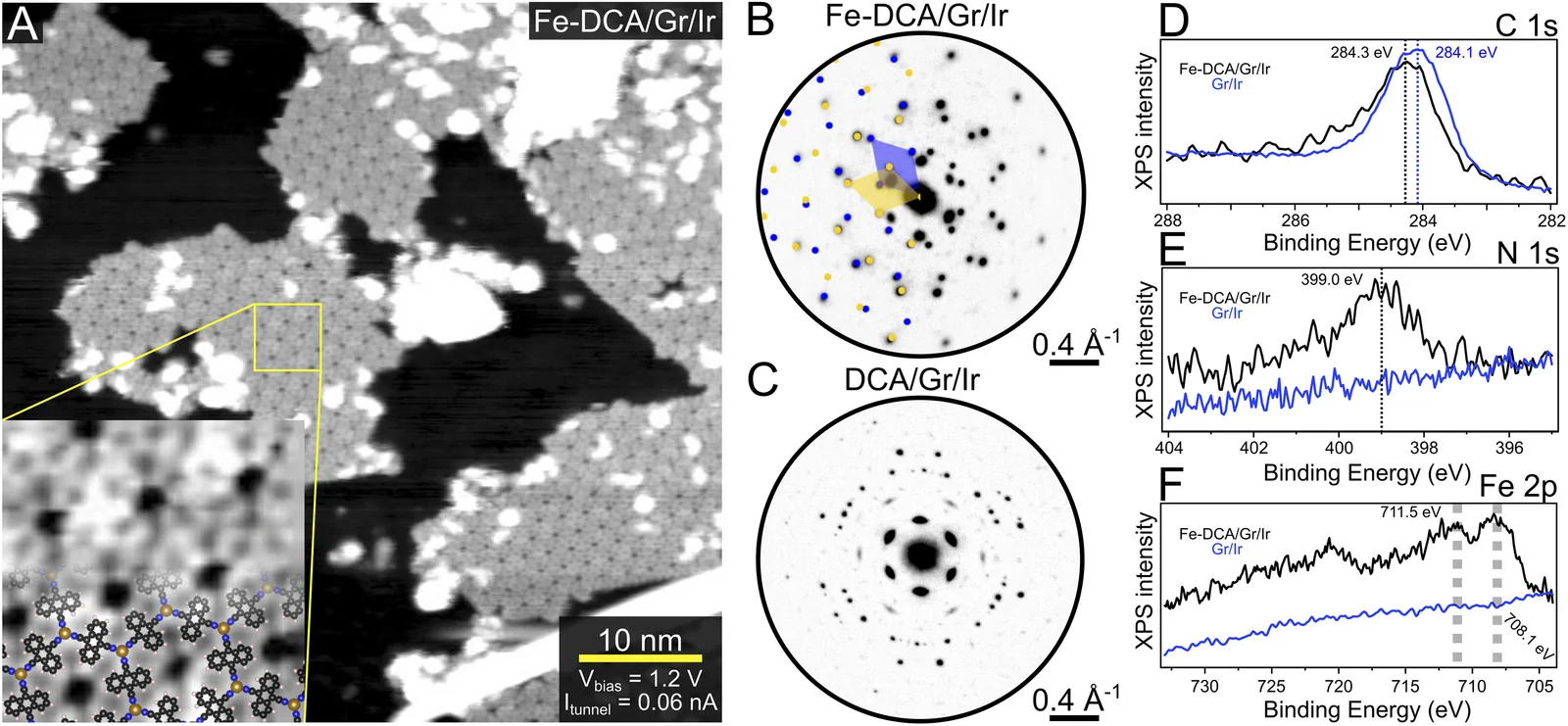
Experimental characterization of Fe-DCA/Gr/Ir(111). (A) STM image of Fe-DCA/Gr/Ir(111). The inset in the lower-left corner shows a zoomed-in area overlaid with a DFT model, showing that Fe-DCA adopts a honeycomb-kagomé lattice with Fe_2_(DCA)_3_ stoichiometry. (B) LEED pattern of Fe-DCA/Gr/Ir(111) is modeled by two mirror-symmetric domains (marked blue and yellow), whose spots are rotated by ∼12° with respect to the graphene moiré spots. Data acquired at a primary electron energy of 18 eV. (C) LEED pattern corresponding to self-assembled DCA on Gr/Ir(111), acquired at a primary electron energy of 16 eV. (D–F) C 1s, N 1s, and Fe 2p regions of XPS spectra acquired on the Fe-DCA/Gr/Ir(111) (black) and clean Gr/Ir(111) (blue).

Fig. 2A shows a room-temperature STM image of Fe-DCA synthesized on Gr/Ir(111) according to the protocol described in the Methods section. Briefly, the graphene surface was first saturated with DCA molecules at ∼80 °C; subsequently, Fe and DCA were co-deposited at the same temperature, followed by post-annealing to ∼80–110 °C to remove excess DCA molecules from the graphene surface. This protocol resulted in the formation of 2D Fe-DCA islands with a characteristic size of tens of nm, covering up to ∼60–70% of the graphene surface. The Fe-DCA framework remains stable upon annealing up to ∼110 °C and, in ultrahigh vacuum, survives for more than two weeks (see Fig. S1 and S3).

The structure of the Fe-DCA network is well-resolved in STM, as shown in the zoomed-in inset in Fig. 2A. The overlaid model is consistent with the previously reported honeycomb-kagomé lattice for metal-DCA 2D MOFs.^5,14,23,24^Fig. 2B shows the long-range periodicity of Fe-DCA observed by low-energy electron diffraction (LEED). The diffraction pattern of Fe-DCA is clearly distinguishable from the pattern of the metal-free molecular phase formed by self-assembled DCA on Gr/Ir(111), shown in Fig. 2C. The diffraction pattern in Fig. 2B can be modeled by the Fe-DCA structure present in two mirror domains, rotated by ∼12° relative to the principal directions of the underlying graphene. The size of the hexagonal Fe-DCA unit cell vector was found to be *a* = *b* = (1.98 ± 0.10) nm; this value is consistent with DFT computations of free-standing Fe-DCA (see Fig. S4).

**Fig. 3:**
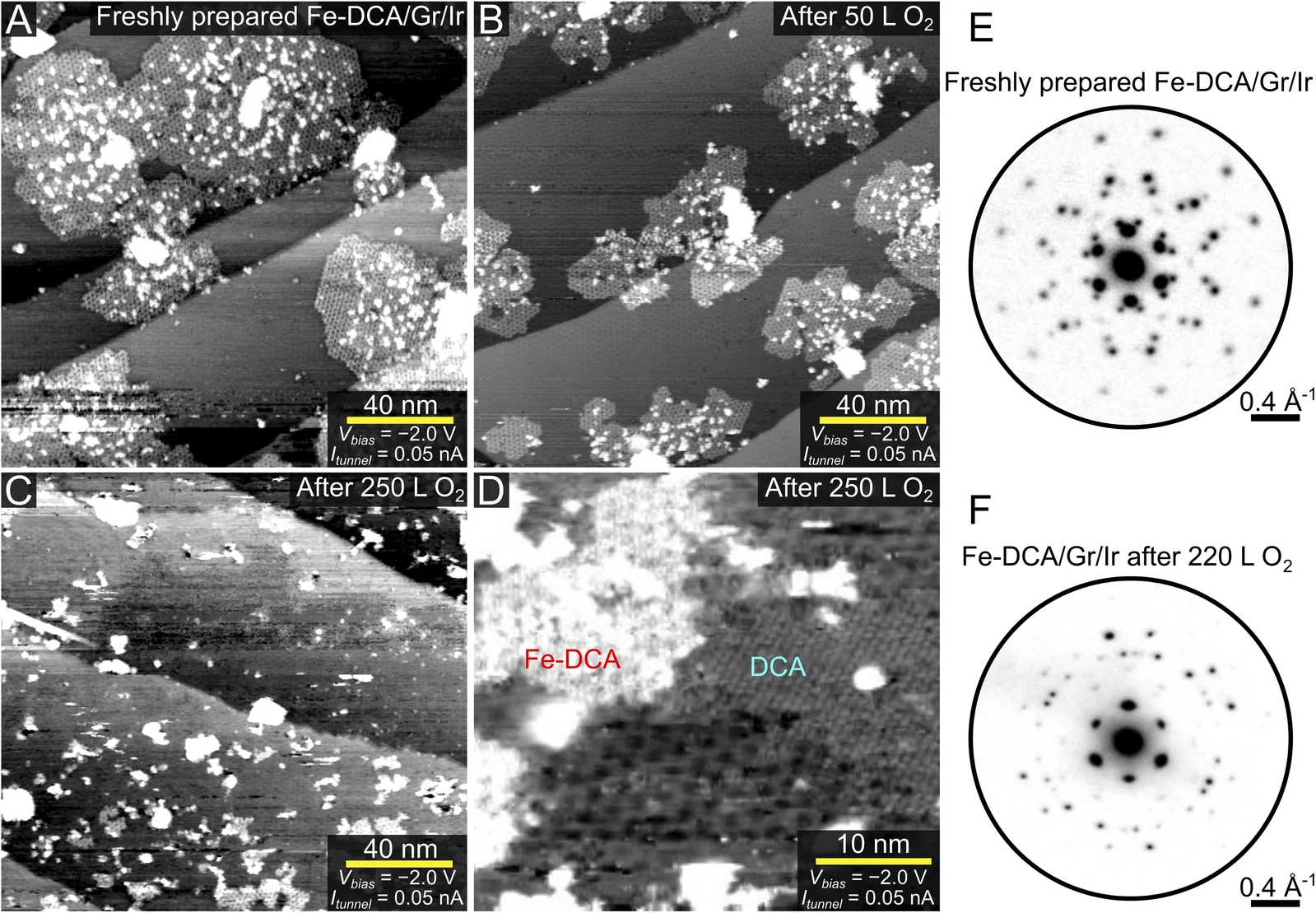
Fe-DCA/Gr/Ir(111) exposed to molecular oxygen. (A–D) STM images of Fe-DCA/Gr/Ir(111) prior to O_2_ exposure (A), after 50 Langmuir (L) O_2_ exposure (B), and after 250 L O_2_ exposure (C and D). The area of Fe-DCA decreases with increasing O_2_ exposure, and islands of self-assembled DCA form (D). (E and F) LEED patterns of freshly-prepared Fe-DCA (E) and Fe-DCA after 220 L O_2_ exposure (F), acquired at 18 eV and 17 eV, respectively. Prior to the exposure, LEED data indicate the presence of Fe-DCA and the absence of self-assembled DCA. After the exposure, a pattern corresponding to self-assembled DCA is observed, while the Fe-DCA pattern is absent.

In addition to the pristine honeycomb-kagomé Fe-DCA structure, our STM data also reveal numerous bright features atop the Fe-DCA layer. Some of these features are visible in Fig. 2A and 3A and B; detailed images are shown in Fig. S2.1. Based on the appearance and specific positions of these features atop the Fe-DCA layer, we interpret many of them as additional DCA molecules bridging neighboring Fe–N_3_ sites within the honeycomb-kagomé Fe-DCA structure (see Fig. S2.1 and S2.2 for details about this assignment). Small variations in the Fe-DCA synthesis protocol (evaporation rates of DCA and Fe, and sample temperature during co-deposition or post-annealing) led to some variations in the local surface density of these species, but no clear trend was identified. Also, no alternative ordered phases of Fe-DCA were observed.

Fig. 2D–F shows the XPS characterization of Fe-DCA/Gr/Ir(111). In the C 1s region, the signal is dominated by the graphene support; the contribution from the DCA molecules appears as a shoulder at the higher-binding-energy side of the graphene C 1s peak. When Fe-DCA is present, the C 1s signal maximum is slightly shifted to higher binding energy by ∼0.2 eV. N 1s signal from Fe-DCA is observed at a binding energy of ∼399.0 eV; the peak position is similar to those measured for self-assembled DCA (see Fig. S6) as well as other molecules featuring cyano groups.^25^ The overall shape of the C 1s and N 1s peaks was similar across different sample preparations, except for intensity variation reflecting differences in surface coverage. The Fe 2p region is generally challenging to interpret due to extensive multiplet splitting.^26,27^ On Fe-DCA/Gr/Ir(111), we observe two signal maxima at ∼708.1 eV and ∼711.5 eV. Based on subsequent gas exposure experiments (described below and in Fig. S5A), we assign the peak at ∼708.1 eV to the Fe bound within Fe-DCA, although we cannot discard the presence of some metallic Fe clusters. The intensity and position of the second signal maximum at higher binding energy vary across different sample preparations (see Fig. S5B), so it is probably not fully related to Fe atoms in the pristine 2D MOF. We speculate that it may be related to some of the bright features observed in STM images, which correspond to Fe atoms with higher coordination and whose coverage varied across different experiments (see Fig. S5).

### Fe-DCA exposed to O_2_

We have exposed the Fe-DCA/Gr/Ir(111) to molecular oxygen to test the ability of Fe–N_3_ sites to adsorb or activate O_2_, as proposed in the literature.^16,28^ When exposed to small doses of molecular oxygen, *i.e.*, <50 L (1 Langmuir (L) corresponds to 1 × 10^−6^ Torr for 1 second), the Fe-DCA islands are still clearly visible in STM but appear significantly smaller (Fig. 3A and B). After a 250 L O_2_ dose, most of the Fe-DCA islands disappear (Fig. 3C), and islands of self-assembled DCA molecules are observed in STM images, co-existing with many disordered small bright clusters of various sizes and shapes (Fig. 3D). LEED data confirm this observation: Fig. 3E and F show the LEED measurements before and after exposure to 220 L O_2_. The diffraction pattern taken after oxygen exposure (Fig. 3F) shows no signatures of Fe-DCA; only the pattern associated with self-assembled DCA is observed (see Fig. 2C for comparison).

**Fig. 4:**
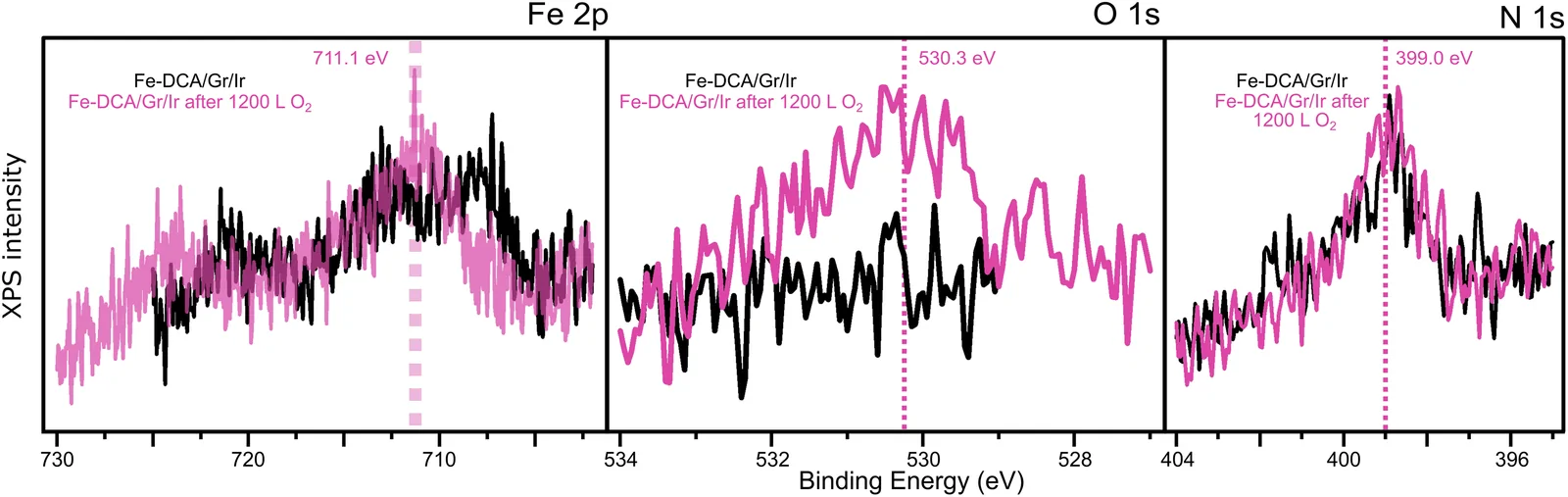
XPS analysis of Fe-DCA/Gr/Ir(111) before (black) and after (pink) exposure to 1200 L O_2_ at room temperature. In the Fe 2p region, the ∼708.1 eV peak disappears upon O_2_ exposure, and a new peak at ∼711.1 eV appears instead. The changes in Fe 2p are accompanied by the appearance of a signal in the O 1s region. The N 1s region appears unchanged, indicating that the DCA molecules did not desorb during the O_2_ exposure.

XPS measured after oxygen exposure is shown in Fig. 4. After very large oxygen doses (1200 L O_2_), a clear O 1s signal appears at ∼530.3 eV; this position is consistent with chemisorbed oxygen^29^ but also with oxygen bound within oxide materials.^30,31^ The N 1s region shows no significant change, indicating that DCA molecules are still present on the surface, in accordance with the STM/LEED data. In the Fe 2p region, significant changes are observed: the previously observed signal maxima are not present anymore, and a single broad Fe 2p_3/2_ peak appears instead at ∼711.1 eV. This position is consistent with oxidized Fe species, suggesting formation of small Fe_*x*_O_*y*_ clusters on the surface.^30,32^

The experimental data thus clearly indicate that the Fe-DCA structure is not stable when exposed to molecular oxygen. The Fe atoms are leached from the Fe–N_3_ coordination nodes to form small Fe_*x*_O_*y*_ clusters, and the liberated DCA linkers reorganize into self-assembled molecular islands.

**Fig. 5:**
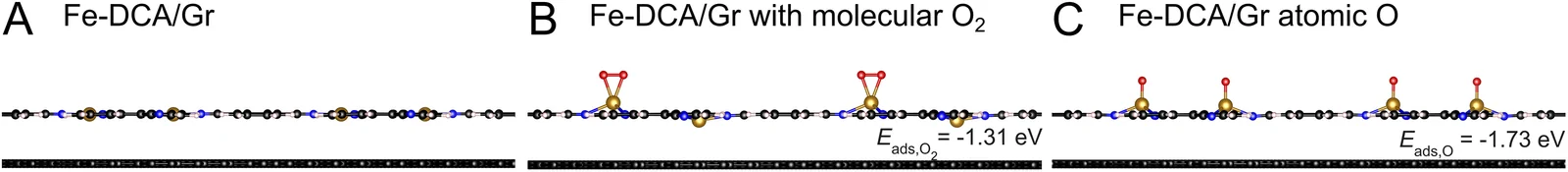
DFT modeling of oxygen adsorption on Fe-DCA/Gr/Ir(111). (A) Pristine structure prior to adsorption. (B) Molecular adsorption of O_2_ at an Fe–N_3_ site. (C) Dissociative adsorption of O_2_ at two neighboring Fe–N_3_ sites (black: carbon, blue: nitrogen, pink: hydrogen, brown: iron, red: oxygen).

Density Functional Theory computations (see Methods for computational details) confirm that molecular oxygen interacts strongly with the Fe–N_3_ sites within Fe-DCA/Gr. First, Fig. 5A shows a DFT-optimized structure of pristine Fe-DCA/Gr, indicating that the graphene-supported structure is almost identical to a free-standing Fe-DCA model in the gas phase (see Fig. 1B and S4). This presents a significant difference from the metal-supported systems, in which the metal atoms are drawn to the support.^14^Fig. 5B shows a DFT-optimized structure with molecular O_2_ being adsorbed at the Fe–N_3_ site; the adsorption energy is −1.31 eV. Finally, Fig. 5C shows a structure with atomic O being adsorbed at two neighboring Fe–N_3_ sites; the adsorption energy (per O_2_ molecule) is −1.73 eV. These ground-state computations cannot capture the structural dynamics leading to the collapse of the Fe-DCA structure, but they clearly indicate that molecular oxygen interacts rather strongly with the Fe–N_3_ sites, and this interaction is stronger than the Fe–N bonding within the MOF (details provided in the SI). Additional insights into a plausible mechanism of Fe-DCA decomposition are provided in Fig. S8 in the SI, revealing that the oxygen molecule can also be dissociated on a single Fe site, inducing the Fe–N bond rupture at a modest energy cost of 150 meV. We hypothesize that a similar mechanism may be responsible for the O_2_-induced decomposition observed in experiments.

### Fe-DCA exposed to CO

Carbon monoxide (CO) is a widely used probe molecule in catalysis research, whose stretching frequency is commonly used to evaluate charge states, coordination numbers, and chemical reactivities of single-atom catalyst sites.^33–35^ Here, we conducted experiments to determine how CO interacts with the Fe–N_3_ sites within Fe-DCA. We first present room-temperature exposure experiments to assess the morphological and chemical changes to Fe-DCA. Further, we present low-temperature STM data that allow us to detect the individual CO molecules adsorbed on the Fe–N_3_ sites.

**Fig. 6:**
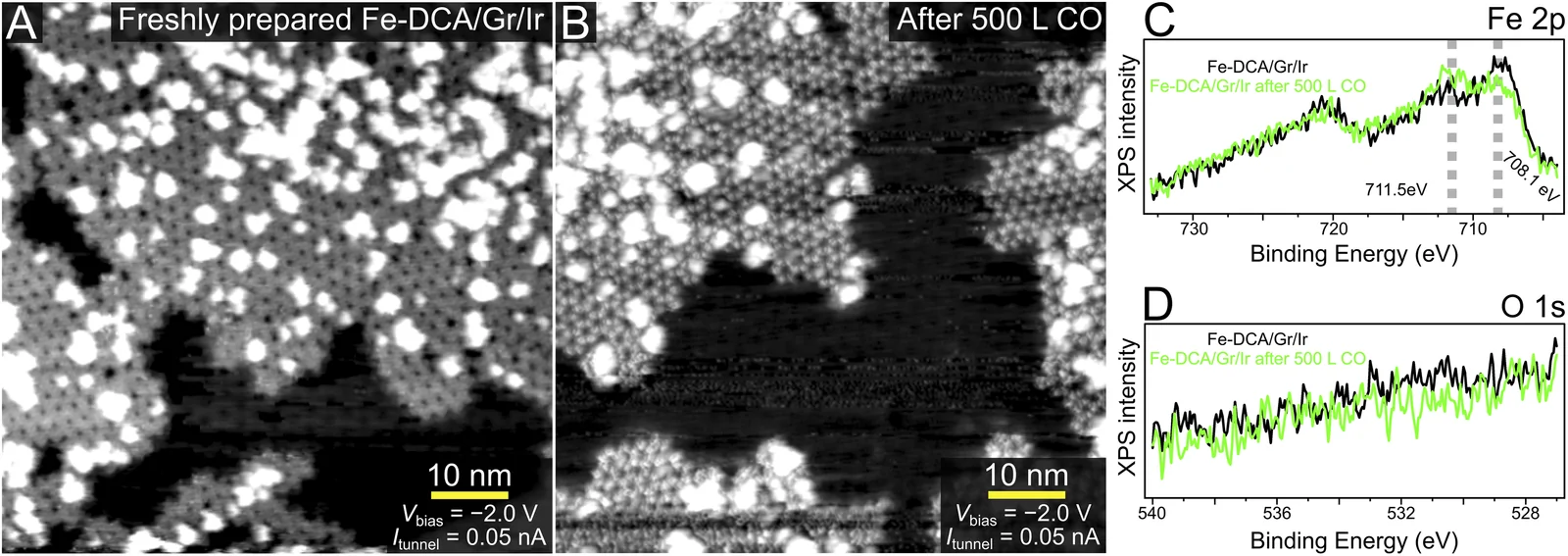
Room-temperature CO dosing on Fe-DCA/Gr/Ir(111). (A) Fe-DCA/Gr/Ir(111) before CO exposure and (B) after 500 L CO exposure. No individual CO molecules and no significant structural changes are observed within the Fe-DCA structure. (C and D) XPS analysis of Fe-DCA/Gr/Ir(111) before (black) and after (green) 500 L CO exposure. The Fe 2p region shows a significant and reproducible change in the relative intensities of components, which indicates that a small fraction of Fe interacted with the CO. (D) No O 1s signal was detected after the CO exposure.

Fig. 6 shows STM and XPS data taken before and after 500 L CO exposure at room temperature. This dataset indicates that the Fe-DCA structure can survive this relatively large CO dose, and we observe no evidence of CO molecules being adsorbed on the individual Fe–N_3_ sites at room temperature. No significant O 1s signal is observed in the XPS spectra, confirming that the amount of adsorbed CO is below the detection limit of the instrument, *i.e.*, significantly lower than the number of Fe–N_3_ sites. However, changes are observed in the Fe 2p region: the intensity of the ∼708.1 eV component is visibly lowered after the CO exposure, while the maximum near ∼711.5 eV is increased. This indicates that a fraction of Fe species chemically interacted with the dosed CO. As the Fe 2p shape does not visibly change upon subsequent annealing at 80 °C, we conclude that CO exposure induced irreversible changes to the Fe-DCA/Gr/Ir(111) sample.

**Fig. 7:**
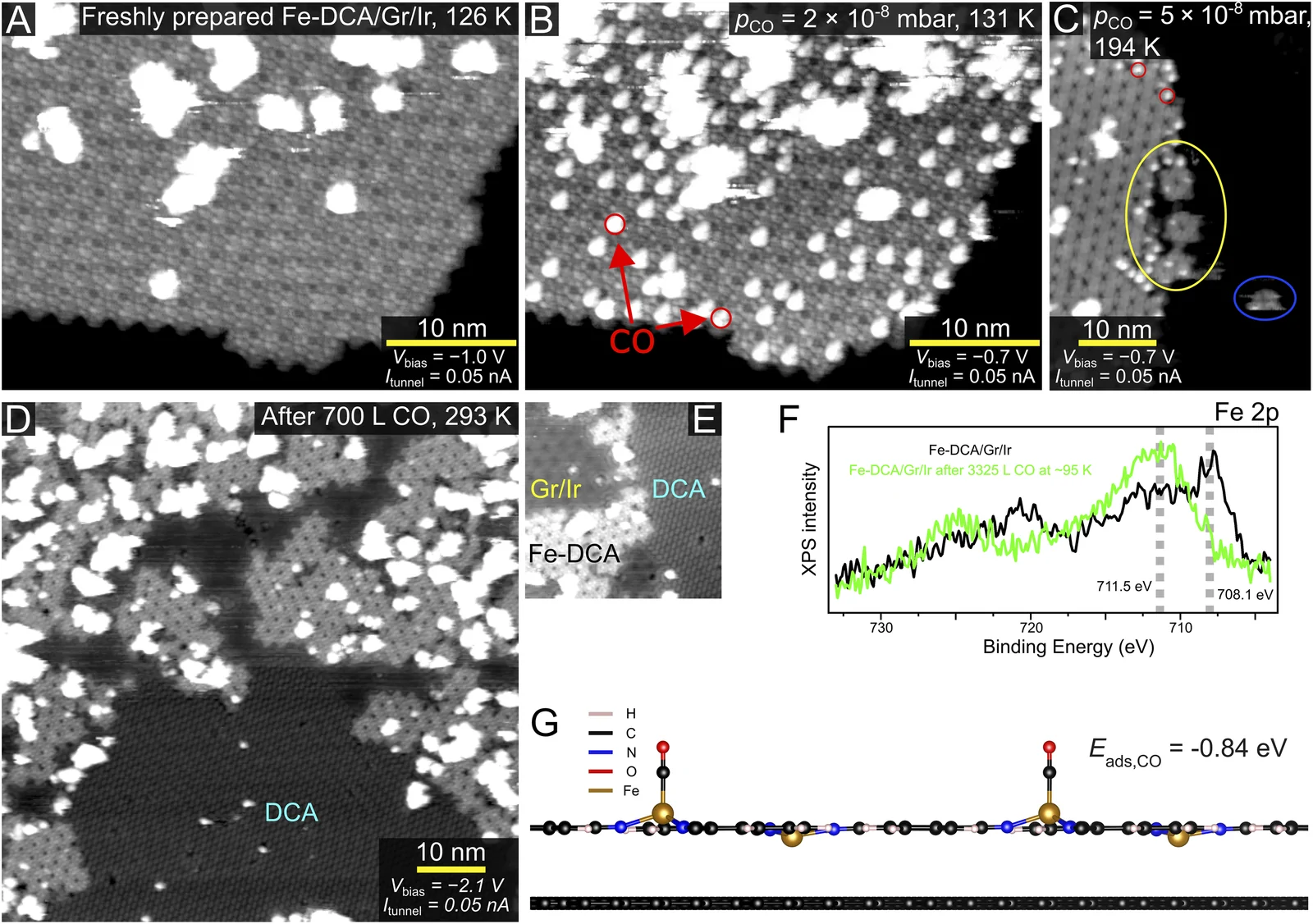
Adsorption of CO molecules on Fe-DCA/Gr/Ir(111) studies by low-temperature STM, XPS, and DFT. (A) STM image of the Fe-DCA/Gr/Ir(111) prior to CO exposure, acquired at 126 K. (B) STM image of the Fe-DCA/Gr/Ir(111) during CO exposure, acquired at 131 K. Representative examples of adsorbed CO molecules, highlighted by red circles. CO dose at the end of the scan is 60 L. (C) STM image acquired during CO exposure at 194 K. The yellow and blue ovals highlight the structural mobility near the Fe-DCA island edges. CO dose at the end of the scan 435 L. (D) After 700 L CO dose at temperatures below 220 K, the Fe-DCA islands are visibly smaller, and islands of self-assembled DCA appear. (E) 30 × 30 nm close-up of the Gr/Ir(111), Fe-DCA, and self-assembled DCA. The acquisition parameters are identical to those in panel D, and the STM image was obtained from the same sample. (F) Fe 2p core level spectra acquired before (black) and after (green) exposure to 3325 L of CO at sample temperatures below 95 K. The ∼708.1 eV component is not present anymore. The XPS measurements took place at room temperature. (G) DFT model of Fe-DCA/Gr with a single adsorbed CO molecule (black: carbon, blue: nitrogen, pink: hydrogen, brown: iron, red: oxygen).

Indeed, repeated CO-exposure experiments at low temperature (Fig. 7A–C) indicate that the CO molecules induce slow but irreversible restructuring at the Fe-DCA island edges, which can eventually lead to a structural collapse of the Fe-DCA network. Fig. 7A shows Fe-DCA/Gr/Ir(111) imaged at 126 K before CO exposure, showing a well-resolved area of pristine Fe-DCA. Fig. 7B shows the same area imaged in the CO background of 2 × 10^−8^ mbar (total CO dose about 60 L). The adsorbed CO molecules are imaged as bright species residing at the Fe–N_3_ sites, both within the Fe-DCA islands and near the island edges. When the temperature is increased to 194 K, the adsorbed CO molecules are found almost exclusively near the lattice defects and edges of the Fe-DCA islands, even when the CO background pressure is increased (5 × 10^−8^ mbar, total dose ∼430 L). Moreover, significant mobility of the Fe-DCA island edges is observed, with some CO_*x*_/Fe_*y*_(DCA)_*z*_ species detaching from the islands (highlighted by yellow and blue ovals in Fig. 7C). Finally, Fig. 7D and E show STM images of the same sample heated to room temperature (following a total dose of >700 L CO at temperatures below 220 K), indicating that the islands of well-defined honeycomb-kagomé Fe-DCA structure are significantly reduced in size, and are found co-existing with a large number of self-assembled DCA molecules. XPS spectra taken after very large CO doses (∼3300 L) at temperatures below 95 K reveal that the Fe 2p component at ∼708.1 eV disappears; this disappearance is correlated to the absence of well-ordered Fe-DCA MOF. We note that no similar CO-induced change in the Fe 2p region is observed when only metallic Fe clusters are present on the surface (see Fig. S5A); this blank experiment thus allows us to assign the ∼708.1 eV component observed on Fe-DCA samples to the Fe species within the MOF structure.

DFT results shown in Fig. 7G indicate that CO adsorbs on the Fe–N_3_ sites within the well-ordered Fe-DCA islands with an adsorption energy of −0.84 eV. This is consistent with the fact that the CO molecules are not stably adsorbed at room temperature, but can be detected at temperatures below 200 K. The CO adsorption energy is also significantly lower than the calculated strength of Fe–N bonding within a perfect Fe-DCA layer, which is consistent with the relative stability of Fe-DCA structure within the 2D MOF islands. Additional DFT computations presented in Fig. S8 reveal that the Fe-DCA decomposition could, in principle, be driven by adsorption of a second CO molecule on the same Fe site. However, the resulting structure is, at experimental conditions, ∼890 meV less stable than the intact Fe-DCA layer, making this decomposition intermediate highly unlikely within the Fe-DCA islands. Nevertheless, we hypothesize that the activation energy required for Fe–N bond rupture can be significantly lower near island edges, where the Fe cations and DCA linkers are undercoordinated and their structural flexibility is higher. A conceptually similar mechanism could therefore be responsible for the slow Fe-DCA disintegration observed experimentally near the Fe-DCA island edges.

## Discussion

We studied the stability and chemical reactivity of Fe-DCA metal–organic frameworks that feature Fe atoms in Fe–N_3_ coordination. Our data show that Fe-DCA on graphene forms a honeycomb-kagomé lattice, similar to that observed in metal-DCA systems on metal supports.^14^ However, in contrast to metal-supported Fe-DCA, our graphene-supported system always featured a significant number of DCA molecules adsorbed atop the 2D MOF. We hypothesize that this may be a consequence of the fact that the local geometry of the Fe–N_3_ sites differs between the two supports: the Fe cations are sunk down on Au(111), whereas on graphene, they are stable in a trigonal-planar geometry. Thus, the higher steric accessibility of the trigonal-planar sites likely facilitates easier complexation of additional DCA molecules. The thermal stability of the resulting DCA-rich structures (which likely featured Fe cations in 4-fold-coordinated geometries, see Fig. S2.2 in SI) was comparable to that of the pristine Fe-DCA 2D MOF, as annealing to 110 °C did not lead to a significant decrease in their number.

Gas exposure experiments and computations reveal that molecular oxygen can be activated on the Fe-DCA network, but this is accompanied by a structural collapse of the Fe-DCA lattice. Carbon monoxide was found to stably adsorb on the Fe–N_3_ sites at temperatures below 200 K. However, higher CO doses induce mobility at the Fe-DCA island edges, leading to the eventual disintegration of the Fe-DCA 2D MOF. These results show that atomically-defined Fe–N_3_ sites within Fe-DCA can be highly reactive, but the limiting factor is the stability of the 2D MOF lattice. An interesting question thus arises whether this structural instability is intrinsic to metal-N_3_ coordination or to the metal-DCA structures, and whether this could be improved by using a different ligand or metal. In this regard, a suitable comparable system is a Fe-PBP 2D MOF,^22,36^ which features Fe–N_3_ sites formed by different nitrogen species, namely pyridinic and pyrimidinic nitrogen. Remarkably, literature indicates that the Fe-PBP/Au(111) system is stable not only in vacuum, but even in an electrochemical environment during oxygen reduction reaction.^36^ This means that Fe–N_3_ sites can be highly stable in realistic conditions, but an open question remains whether this stability is intrinsic to the Fe-PBP MOF itself, or whether it is induced by the rather strong interaction with the Au(111) support.^22^ To answer this question, more studies on chemically inert supports are needed, which will quantify the effects arising from 2D MOF-support interactions,^11,20^ and thus allow for the quantification of the intrinsic stability and reactivity of single-atom sites embedded within supported 2D materials.

## Conclusions

Our work shows that Fe-DCA can be reproducibly prepared on graphene and form well-ordered 2D MOFs with a defined honeycomb-kagomé lattice. This structure is thermally stable up to ∼110 °C but shows only limited stability against gas exposure. Room-temperature adsorption of molecular oxygen causes rapid structural collapse of the Fe-DCA lattice, leaving Fe_*x*_O_*y*_ clusters and self-assembled DCA linkers on the surface. The effect of carbon monoxide exposure is less dramatic, but CO molecules induce local rearrangement of the Fe-DCA lattice near the island edges, which consequently leads to slow disintegration of the Fe-DCA 2D MOF. Stable adsorption of CO on the Fe–N_3_ sites can be observed at temperatures below 200 K. Taken together, our results establish metal-DCA systems on graphene as suitable systems for probing the intrinsic reactivity of metal-N_3_ sites and simultaneously outline limitations for using these systems in realistic conditions.

## Methods

Experiments were carried out in an ultrahigh vacuum system consisting of multiple chambers interconnected by a central transfer line separated by gate valves. The base pressure of all the chambers used in this study is below 5 × 10^−10^ mbar. The Ir(111) single crystals (supplied by MaTecK and SPL) were cleaned by cycles of Ar^+^ sputtering (1.5 keV, 10 min) and followed by annealing to 1300 °C (10 min). When graphene was present on the sample prior to cleaning, the first annealing cycle took place in O_2_ background (1000 °C, *p*_O_2__ = 1 × 10^−6^ mbar). The temperature was measured with a LumaSense IMPAC IGA 140 pyrometer with the emissivity set to 0.1.

Graphene on Ir(111) was grown by adsorbing saturation coverage of ethylene at room temperature, followed by pumping out the ethylene background and ramping the temperature up to 1250 °C in ultrahigh vacuum. Then, the sample was re-exposed to ethylene at this temperature (1 × 10^−6^ mbar, 5 min). This protocol combines temperature-programmed growth with chemical vapor deposition and consistently leads to a full monolayer coverage of high-quality Gr/Ir(111).^37^

On the resulting Gr/Ir(111) substrates, Fe-DCA 2D MOF was synthesized. DCA molecules were evaporated from a crucible heated in a heat transfer fluid bath; the temperature of the heat transfer fluid was 110 °C. Fe was deposited from an effusion cell heated to 1030 °C (deposition rate ∼11.5 fm s^−1^). The metal deposition rate was calibrated by a water-cooled quartz microbalance. The synthesis protocol comprised pre-saturating the graphene substrate with DCA at ∼70–80 °C, followed by co-deposition of the metal and DCA at the same substrate temperature. In some preparations, samples were subsequently kept at ∼80–110 °C for an additional 10–20 min. However, annealing did not systematically improve the quality of these 2D MOF (see Fig. S1); thus, it was not applied to all syntheses.

Scanning tunneling microscopy images were recorded in constant-current mode using a commercial system (Aarhus 150, SPECS) using a tungsten tip. Distortion in the STM images was corrected to fit the known dimensions of Gr/Ir(111) moiré unit cell. Where possible, nonlinear image distortion was corrected as described in ref. 38. Low-energy electron microscopy/diffraction experiments were carried out in a SPECS FE-LEEM P90 instrument, the LEED data were acquired using microdiffraction apertures of sizes 100 µm and 50 µm,^39^ and the LEED models were done in the ProLEED software.^40^ XPS was performed on a SPECS system utilizing a Phoibos 150 spectrometer. All measurements employed a non-monochromatized X-ray source (Mg Kα, *hν* = 1253.6 eV) with an emission angle of 70° and high-magnification mode (magnification = 10, acceptance angle ±9°). The pass energy was set to 30 eV for C 1s, 50 eV for N 1s and O 1s, and 90 eV for Fe 2p, all with a dwell time of 0.1 s. The photoelectron spectra were smoothed using tools implemented in SpecsLab Prodigy v4.114.1, namely the noise estimation algorithm with a smoothing parameter set to −1. Subsequently, the spectra were normalized to the low binding energy background.

Spin-polarized density functional theory (DFT) calculations were carried out using the Vienna *ab initio* Simulation Package (VASP).^41^ Core electrons were treated with the projector augmented-wave (PAW) method.^42^ Exchange–correlation effects were described using the nonlocal van der Waals-corrected optPBE-vdW functional.^43^ A Hubbard-type on-site correction of *U* − *J* = 4 eV within Dudarev's approach^44^ was applied to properly capture the Fe 3d states. For Fe, N, H, O, and C, 8 valence electrons (4s^2^3d^6^), 5 valence electrons (2s^2^2p^3^), 1 valence electron (1s^1^), 6 valence electrons (2s^2^2p^4^), and 4 valence electrons (2s^2^2p^2^), respectively, were expanded in a plane-wave basis with a kinetic energy cutoff of 520 eV. Brillouin-zone integrations employed a *Γ*-centered 3 × 3 × 1 Monkhorst–Pack grid.^45^ Structural relaxations were stopped when residual forces acting on all atoms were below 0.02 eV Å^−1^.

For the interface calculations, a single graphene sheet was considered as the substrate. Interface models were constructed by fixing the substrate atoms at their DFT-relaxed positions, aligning the molecular layer accordingly, and introducing a 15 Å vacuum region. The Fe-DCA/Gr interface was modeled using the graphene supercell with the matrix notation 
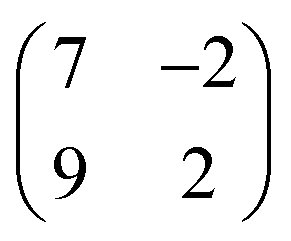
 combined with the primitive cell of the honeycomb-kagomé Fe-DCA layer. The resulting interface supercell lattice constants are 0.8% larger than those of the gas-phase Fe-DCA model.

## Author contributions

D. H., Z. J., T. L., and A. J. acquired and analyzed the experimental data; J. P. and T. L. carried out the computational analysis. Z. J. and J. Č. conceptualized and supervised the research. Z. J. and J. Č. acquired the research funding. D. H. and Z. J. wrote the manuscript with the input from all co-authors.

## Conflicts of interest

There are no conflicts to declare.

## Supplementary Material

RA-OLF-D6RA03820D-s001

## Data Availability

The raw data associated with this study are deposited in the Zenodo repository [DOI: https://www.doi.org/10.5281/zenodo.21495250]. Supplementary information (SI): additional STM, XPS and DFT data. See DOI: https://doi.org/10.1039/d6ra03820d.
