# Supplementary material for: Stability and reactivity of Fe-DCA 2D metal–organic frameworks on chemically inert graphene support
Source: RSC Adv. 2026 Sep 24;16(45):57232–40. doi: 10.1039/d6ra03820d (PMC13603061; doi:10.1039/d6ra03820d)
Supplement: Supplementary file 1 [file RA-016-D6RA03820D-s001.pdf]

Supplementary Information (SI) for RSC Advances.
This journal is © The Royal Society of Chemistry

Supplementary Information

Stability and Reactivity of Fe-DCA 2D Metal-Organic Frameworks on Chemically Inert Graphene Support

Dominik Hrůza [1], Jakub Planer [1], Tadeáš Lesovský [1], Ayesha Jabeen [1], Jan Čechal [1,2*], Zdeněk Jakub [1*]

[1] CEITEC - Central European Institute of Technology, Brno University of Technology, Purkyňova 123, 612 00 Brno, Czech Republic.

[2] Institute of Physical Engineering, Brno University of Technology, Technická 2896/2, 616 69 Brno, Czech Republic.

Correspondence to: cechal@fme.vutbr.cz, zdenek.jakub@ceitec.vutbr.cz

Table of Contents

1 Temperature stability and domain orientation of Fe-DCA *1*

2 Bright features atop Fe-DCA *2*

3 Stability of Fe-DCA over time *3*

4 DFT model of the free-standing Fe-DCA and graphene-supported Fe-DCA *4*

5 Additional XPS measurements of the Fe 2p region *5*

6 Additional XPS measurements of the self-assembled DCA molecules *6*

7 Stability of Fe-DCA in Argon atmosphere *6*

8 Plausible intermediates for the adsorption-induced decomposition mechanism *7*

1 Temperature stability and domain orientation of Fe-DCA

For Fe-DCA, no significant improvement of the 2D MOF quality was found after annealing in the 90–110 °C range. Heating to 130 °C resulted in an almost complete collapse of the Fe-DCA 2D MOF, as shown in Figure S1.

The LEED data presented in Figure 2B of the main text reveal that the Fe-DCA structure forms two mirror domains, each rotated by about ~12° relative to the underlying graphene unit cell. These findings are confirmed by the STM observations in Figure S1A, where the pink-highlighted domain is shown to be rotated by ~24° with respect to the yellow domain.

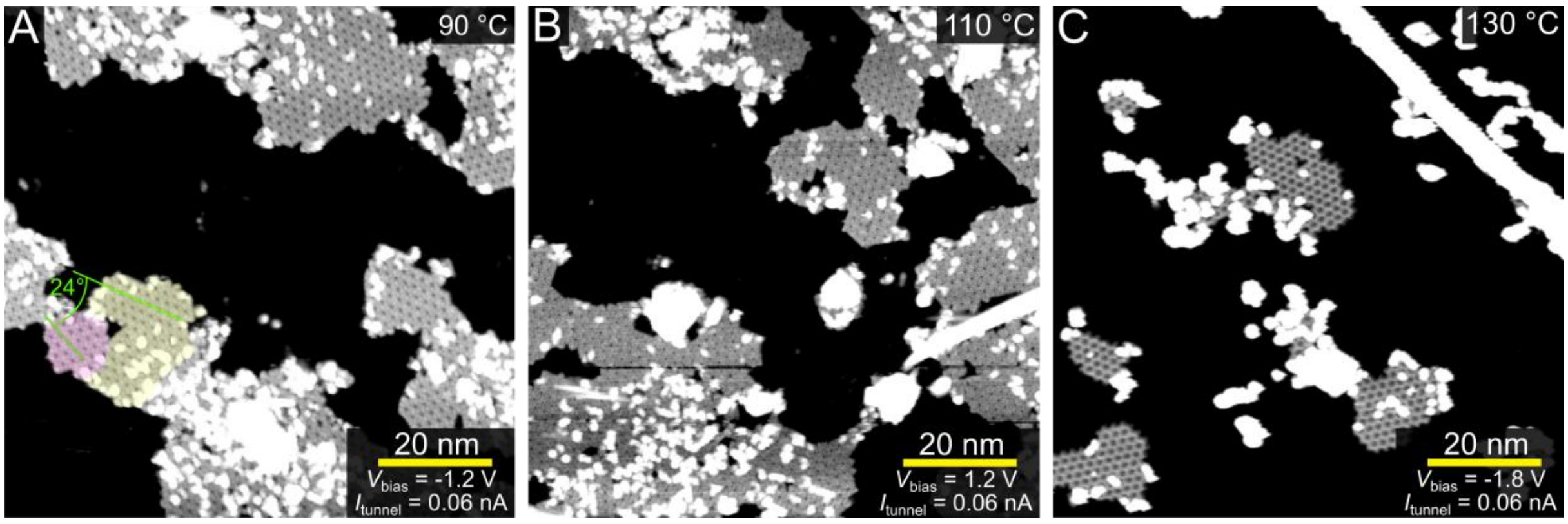

Figure S1: Thermal stability of Fe-DCA/Gr/Ir(111). (A) Fe-DCA after annealing at 90 °C. In the lower left part of the image, the domains highlighted in pink and yellow enclose an angle of ~24°. (B) Fe-DCA after a repeated annealing step at 110 °C, showing no significant changes in coverage compared to image A. (C) Fe-DCA after annealing at 130°C, where most of the 2D MOF decomposed. The annealing time was identical for all experiments (20 min).

2 Bright features atop Fe-DCA

As described in the main text, additional bright features were consistently observed atop Fe-DCA/Gr/Ir(111) samples, shown in Figure S2.1. Most of these features have a specific position and appearance, as shown in the inset (1-4) in Figure S2.1. The characteristic elongated shape of these features is located midway between two Fe-N_3 sites of the Fe-DCA lattice, and these features can be found in three symmetry-equivalent orientations of the underlying Fe-DCA lattice. Some of the bright features are found isolated (as highlighted by the yellow circles in Figure S2.1), but more often they reside close to each other, forming larger "clusters". When three of the individual features reside around the same Fe-N_3 site, a characteristic "clover" shape is observed, as highlighted by the pink circles in Figure S2.1B, and in the inset (5-6). These observations support the interpretation that most of these individual bright features are DCA monomers coordinated to the Fe-N_3 sites within the Fe-DCA 2D MOF. This assignment is further supported by additional DFT calculations presented in Figure S2.2A, showing the optimized geometry of DCA monomer residing atop Fe-DCA/graphene. The adsorption energy of the DCA monomer is −1.72 eV, which is consistent with the presence of DCA monomers in experimental STM images acquired at room temperature. The simulated STM image in Figure S2.2B reveals the characteristic appearance and positioning of the additional DCA molecule: the highest intensity is located at the anthracene body of the molecule, while the cyano groups anchoring the DCA molecule are visible with slightly lower intensity. This is in very good agreement with the experimental data shown in insets 1-4 within Figure S2.1.

We note that the Fe cations within the DCA/Fe-DCA structure are fourfold coordinated, and their coordination geometry is nonplanar, as the Fe atoms are lifted above the Fe-DCA monolayer. The experimentally observed tendency of the DCA monomers to form “clusters” can be intuitively explained by this phenomenon: the lifted Fe atoms are likely to be more accessible for further complexation with excess DCA. Thus, placing two or three DCA molecules around the same Fe atom is likely to result in more stable structures; and such structures are indeed observed in experiments.

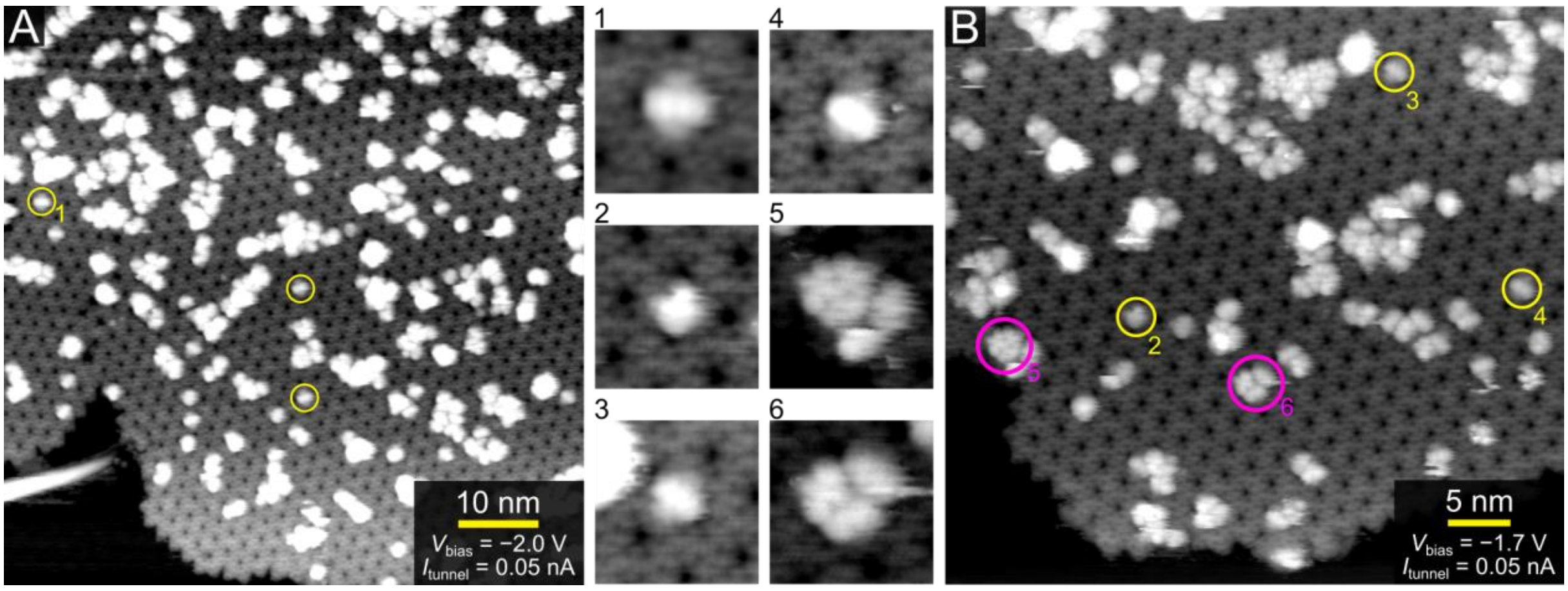

Figure S2.1: Detailed STM images of the bright features atop Fe-DCA/Gr/Ir(111). Most of the bright species atop Fe-DCA consist of monomers highlighted by yellow circles and shown in the zoomed-in inset (1-4). These species have a characteristic shape and are commonly found to be bridging two neighboring Fe-N_3 sites; therefore, we interpret them as DCA monomers. These monomer species are sometimes found isolated, but more often they reside near each other, leading to the larger “cluster” appearance. When three species are arranged around a single Fe-N_3 site, a characteristic “clover” motif appears, highlighted by pink circles in panel B and in zoomed-in insets (5) and (6). Notably, this “clover” motif is strikingly similar to the Fe-N_3 motif within the Fe-DCA network.

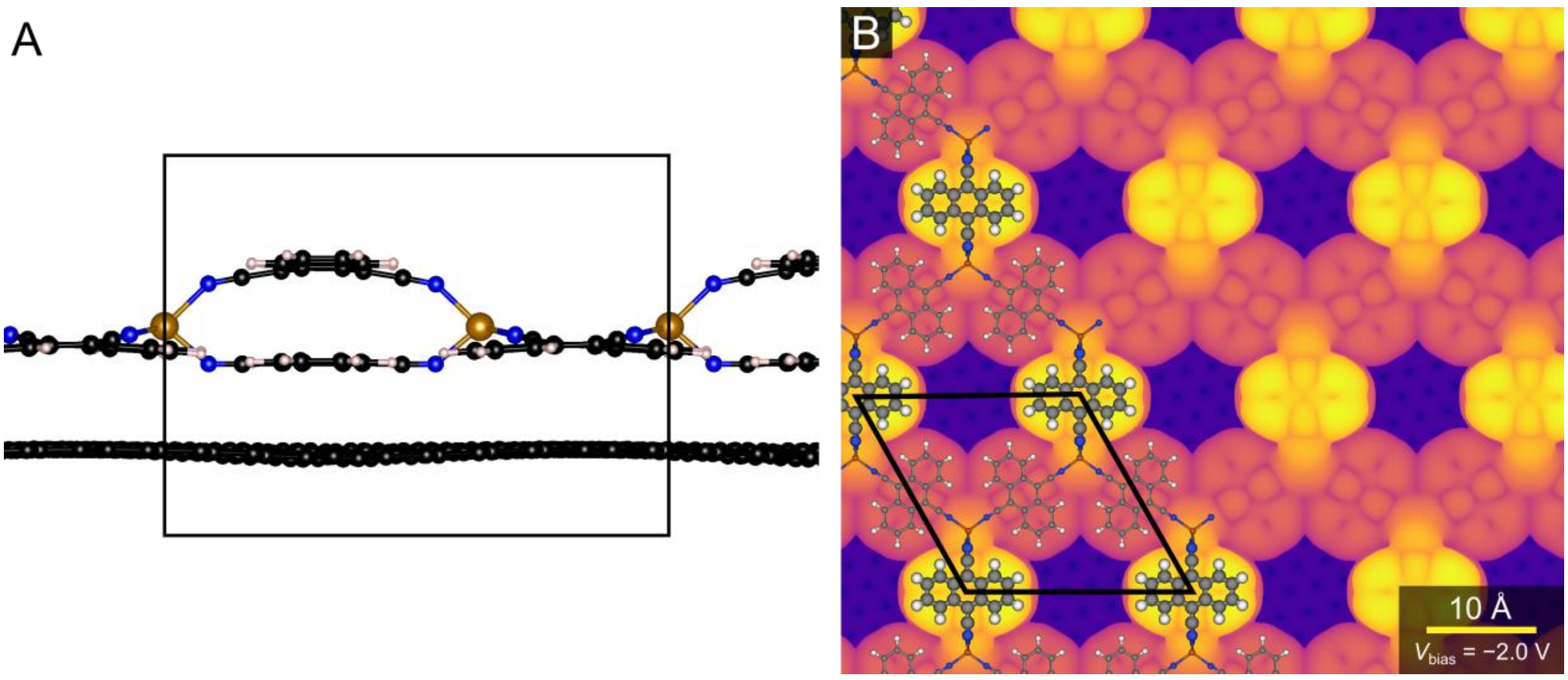

Figure S2.2: Calculated DCA molecule adsorbed atop Fe-DCA. Panels A and B show the side view of the optimized geometry, and the simulated STM image, respectively.

3 Stability of Fe-DCA over time

To verify the intrinsic stability of Fe-DCA/Gr/Ir(111) in the absence of reactive gases, room temperature STM measurements were performed immediately after preparation (Figure S3A)

and again after 16 days of storage in ultrahigh vacuum at a pressure below 5×10^{-10} mbar (Figure S3B). Prior to the second measurement, the sample was annealed to ~110 °C to minimize possible artifacts arising from adventitious adsorbates accumulated during storage. Between Figure S3A and Figure S3B, the Fe-DCA surface coverage decreased from (69 ± 5) % to (62 ± 9) % (mean value ± expanded uncertainty at a 95% confidence level). The coverage was evaluated across 4 different positions within a similar region of the 2 µm × 2 µm scanning window on a freshly-prepared sample *(n = 4)*, and from 5 distinct locations on the sample after 16 days *(n = 5)*. This comparison indicates that the rate of Fe-DCA framework degradation is very slow in the absence of CO or O_2.

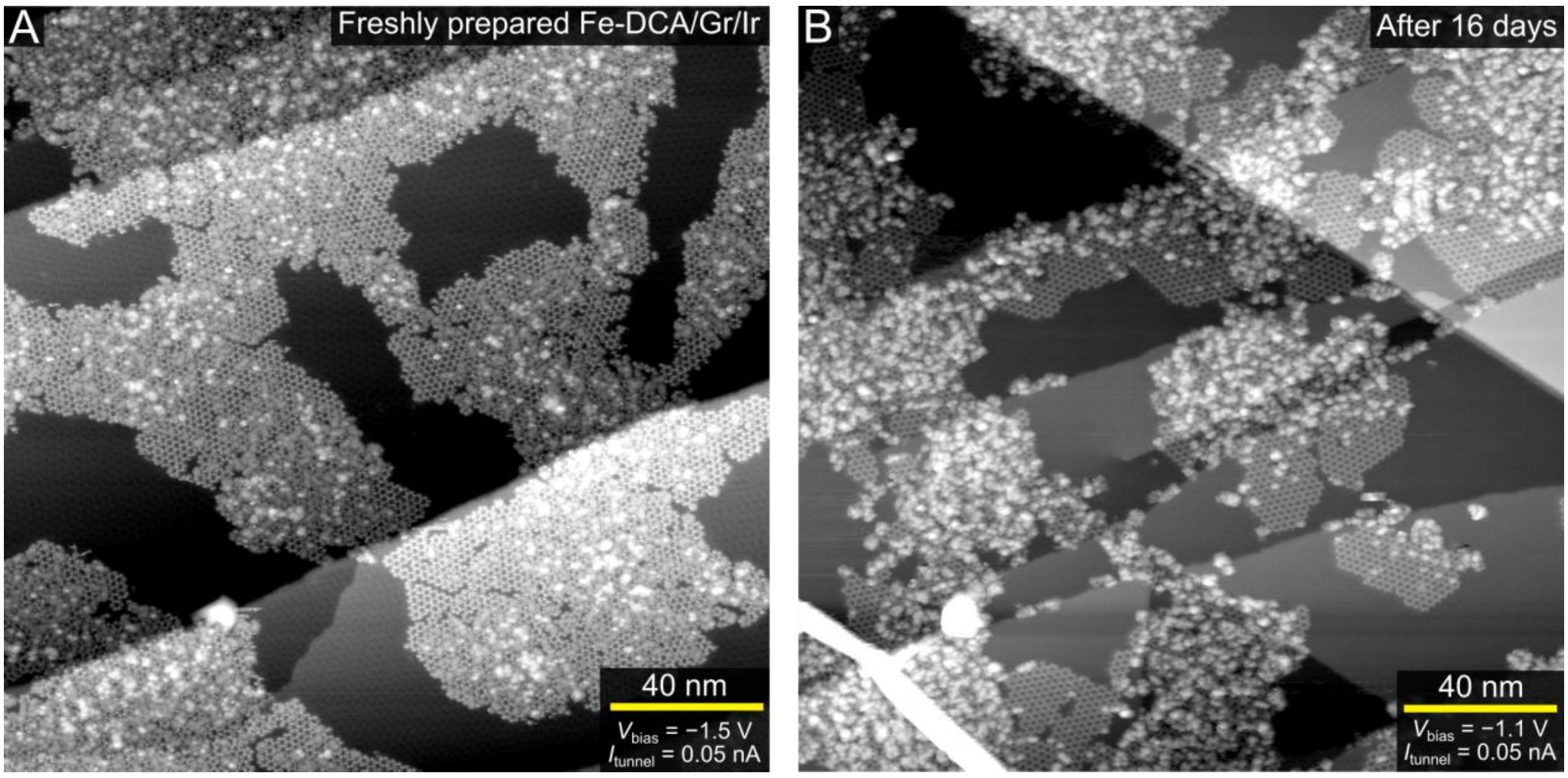

Figure S3: Room-temperature STM images of Fe-DCA/Gr/Ir(111): A) freshly prepared; B) after 16 days in ultrahigh vacuum and annealing at 110 °C.

4 DFT model of the free-standing Fe-DCA and graphene-supported Fe-DCA

According to DFT calculations (see Methods section for computational details), the Fe-DCA structure remains planar on graphene (comparing Figure S4A and Figure S4C), and no significant change in the Fe-DCA lattice parameter is observed (comparing Figure S4B and Figure S4D). The influence of the graphene support is therefore negligible.

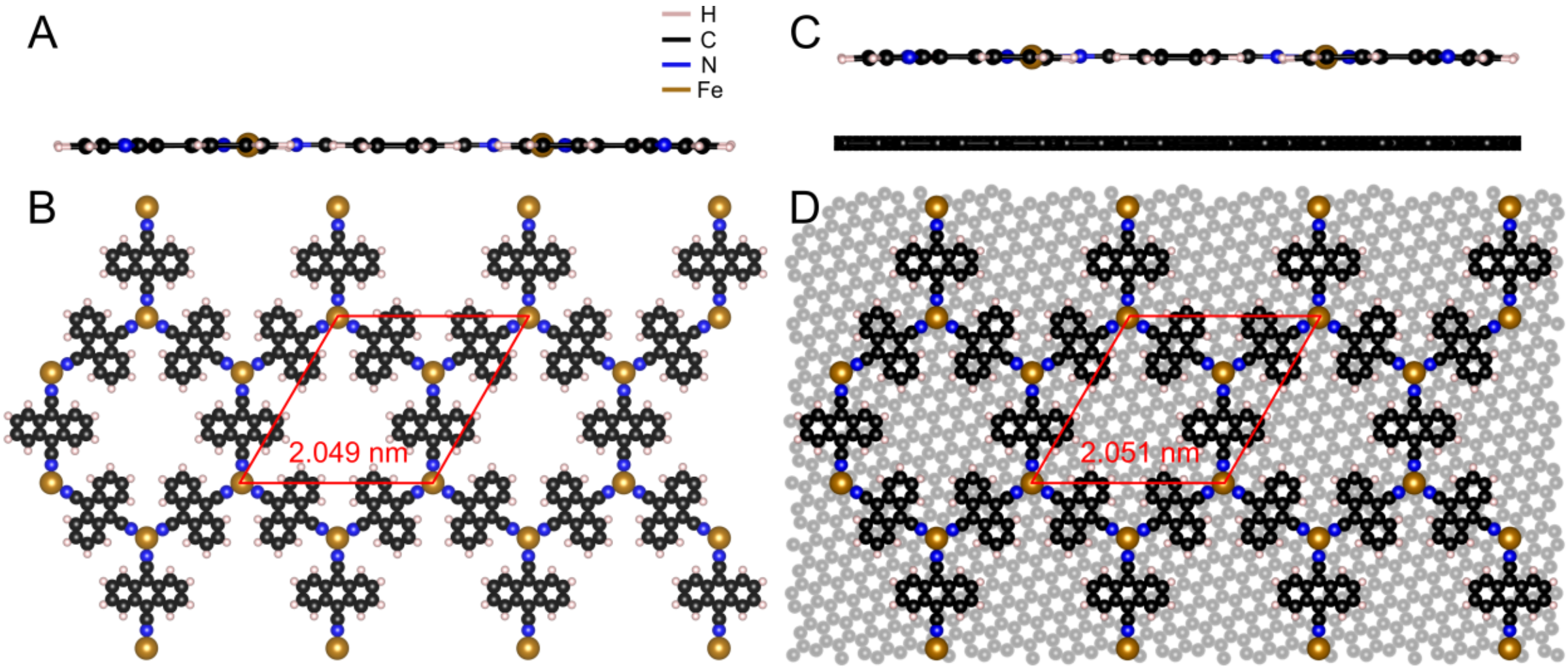

Figure S4: DFT-calculated model of the Fe-DCA honeycomb-kagome structure. Free-standing Fe-DCA panels (A) and (B) and graphene-supported Fe-DCA panels (C) and (D) show the side and top views, respectively. Atoms are shown in black (carbon), blue (nitrogen), pink (hydrogen), and brown (iron). The unit cells are marked in red, with a calculated lattice parameter of 2.049 nm for free-standing Fe-DCA and 2.051 nm for graphene-supported Fe-DCA.

5 Additional XPS measurements of the Fe 2p region

Figure S5A shows the Fe 2p region of Fe-DCA/Gr/Ir(111) compared to Fe 2p region of metallic Fe/Gr/Ir(111) before (gray) and after (green) exposure to 1700 L CO at ~95 K. While the Fe 2p region of the Fe-DCA/Gr/Ir(111) system is significantly changed after the exposure (as shown in Figures 6 and 7 in the main text), no significant change is observed on the spectrum corresponding to metallic Fe clusters. This comparison supports the interpretation that on the Fe-DCA/Gr/Ir(111) sample, most of the signal at ~708.1 eV originates from Fe species within Fe-DCA, and not from Fe clusters.

Figure S5B shows three representative measurements of the Fe 2p region of Fe-DCA/Gr/Ir(111), showing that the intensity and peak position of the ~711.5 eV component significantly varied between sample preparations that were nominally identical.

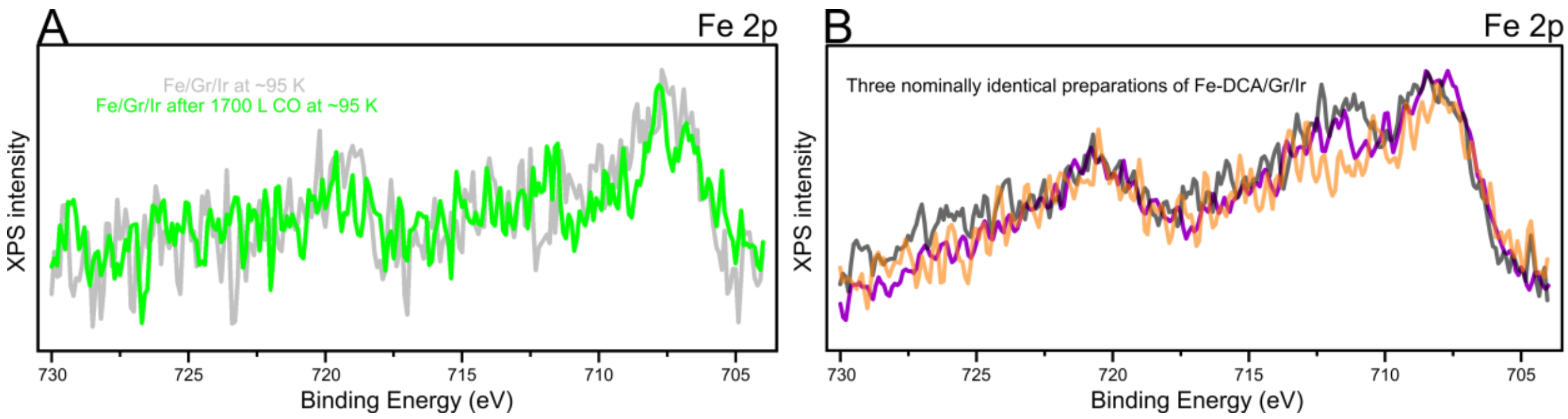

Figure S5: XPS measurements of the Fe 2p region. (A) XPS of Fe/Gr/Ir(111) before (black) and after (green) exposure to 1700 L CO at ~95 K. The Fe 2p line shape did not exhibit major changes. Measurements were carried out at ~95 K. (B) XPS of Fe-DCA/Gr/Ir(111) showing variations in position and intensity of the ~711.5 eV component for three representative Fe-DCA/Gr/Ir(111).

6 Additional XPS measurements of the self-assembled DCA molecules

Figure S6A shows the C 1s region after DCA deposition on Gr/Ir(111) compared to the reference measurement for Gr/Ir(111). The shift in peak position toward higher binding energy is less than 0.1 eV.

Figure S6B compares the N 1s region for DCA and Fe-DCA deposited on Gr/Ir(111).

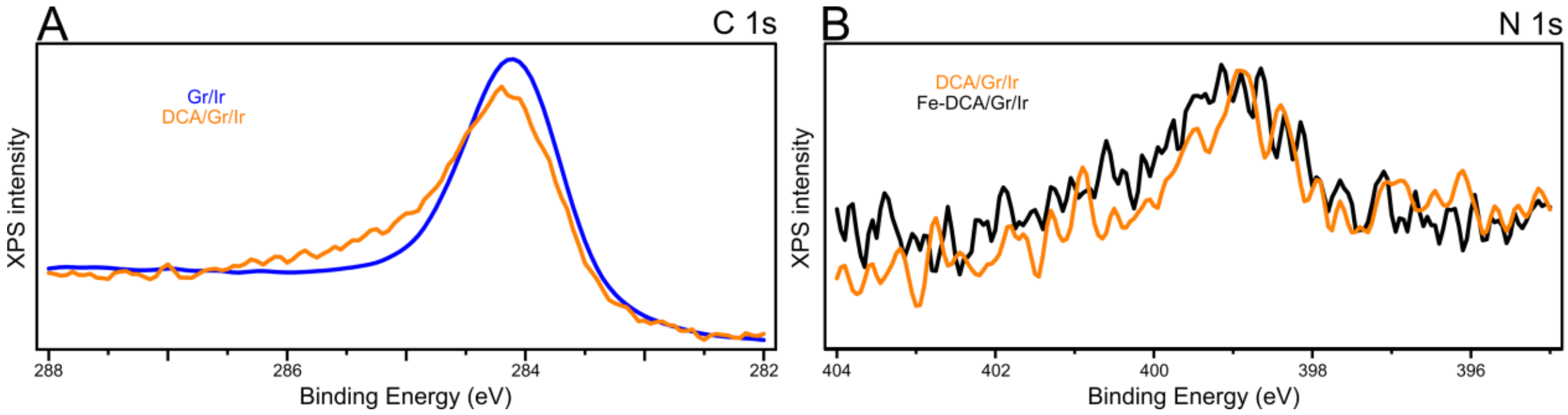

Figure S6: XPS measurements of the C 1s and N 1s region. (A) XPS of C 1s for Gr/Ir(111) (blue) and after DCA deposition on Gr/Ir(111) (orange). (B) XPS of N 1s after DCA deposition on Gr/Ir(111) (orange) and Fe-DCA deposition on Gr/Ir(111) (black). The black spectra correspond to the XPS data presented in Figure 2 of the main text.

7 Stability of Fe-DCA in Argon atmosphere

To verify that the Fe-DCA decomposition observed after high doses of O_2 and CO is really induced by the chemical interaction, and not just by the increased pressure during dosing, we have tested the Fe-DCA stability in an Ar atmosphere. Figure S7 shows room temperature STM measurements performed immediately after preparation (Figure S7A) and again after a 5000 L Ar exposure at the pressure ~3×10^{-6} mbar (Figure S7B). As evident from Figure S7B, the Fe-DCA structure remains stable even after an Ar dose significantly higher than those used for O_2 and CO.

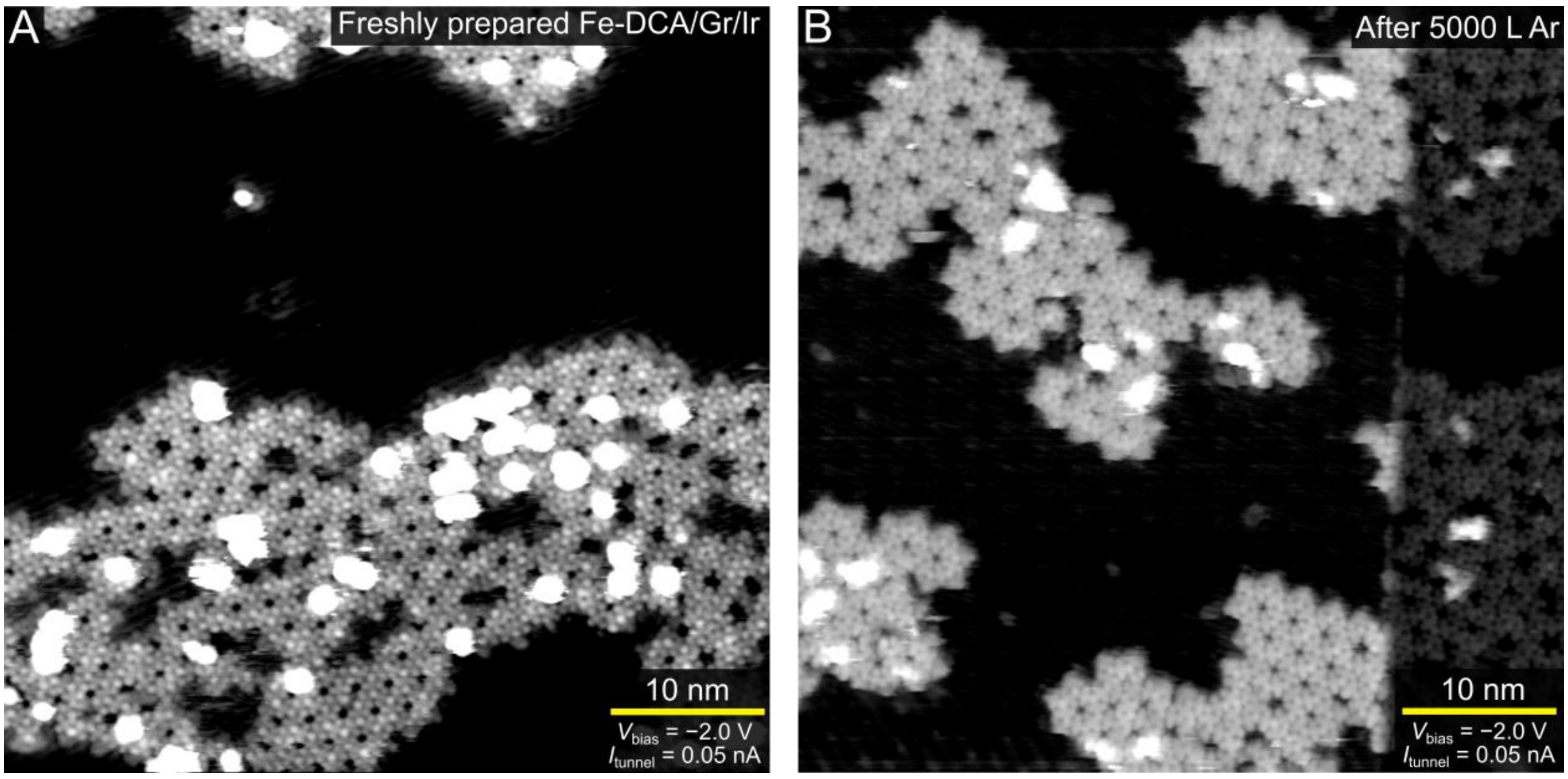

Figure S7: Room-temperature STM images of Fe-DCA/Gr/Ir(111): A) freshly prepared; B) after 5000 L Ar exposure.

8 Plausible intermediates for the adsorption-induced decomposition mechanism

In this section, we discuss a plausible decomposition mechanism in an O_2 gas atmosphere. The first indication of instability comes from comparing the calculated Fe-O_2 binding energy with the bond energy of Fe atom and the carbonitrile group within the Fe-DCA framework. Our calculations show that the strength of the Fe-O_2 and Fe-N interactions are very similar, the difference is only 20 meV: -1.31 eV for Fe-O_2 binding, compared with -1.29 eV for Fe-N bond. This result already suggests that the oxygen bond may effectively replace the Fe-N bond, leading to the decomposition of Fe-DCA. To explore this possibility further, we performed further DFT calculations to identify the possible intermediates of the adsorbate-assisted decomposition process. To keep these calculations simple, the graphene support was removed, which also allows the lattice parameters to relax during the structural optimization.

We have identified a promising intermediate for Fe-DCA decomposition in the O_2 atmosphere, as shown in Figure S8B. Here, an Fe-N bond can be decomposed upon a dissociative adsorption of an oxygen molecule at the same Fe site, at an energy cost of 150 meV relative to the side-on molecular adsorption on Fe-DCA (Figure S8A). We suspect that such decomposition may initiate an irreversible process, especially near the edges of the Fe-DCA network, where the dangling DCA molecules can be separated from the network to form a pure DCA layer. Formation of a pure DCA layer may provide an additional thermodynamic driving force for this process, which would be further stabilized by 461 meV per DCA molecule, exceeding the 150 meV energy penalty associated with dissociative O_2 adsorption and potentially compensating for the additional cost of detaching DCA from the Fe-DCA framework, which was not evaluated explicitly.

Regarding the CO-assisted decomposition, our calculations show that the adsorption of a single CO molecule is not sufficient to cleave the Fe-N bond, as the adsorption energy of CO (-0.76 eV) is much lower than the strength of the Fe-N bond (-1.29 eV). We further tested whether adsorption of multiple CO molecules could induce a similar decomposition mechanism as that induced by dissociated oxygen. Our results, shown in Figure S8D, indicate that such a process would cost 890 meV at experimental conditions ($T = 180$ K, $p = 10^{-8}$ mbar). These results are consistent with the absence of CO-induced decomposition within the Fe-DCA islands observed in experiment. With respect to a similar mechanism happening near the island edges (as observed in the experiment), our computations using periodic boundary conditions cannot explicitly assess the energetics of such a process, but it is expected that the energy barrier would be significantly lowered by the higher flexibility of the undercoordinated cations or linkers near the island edge.

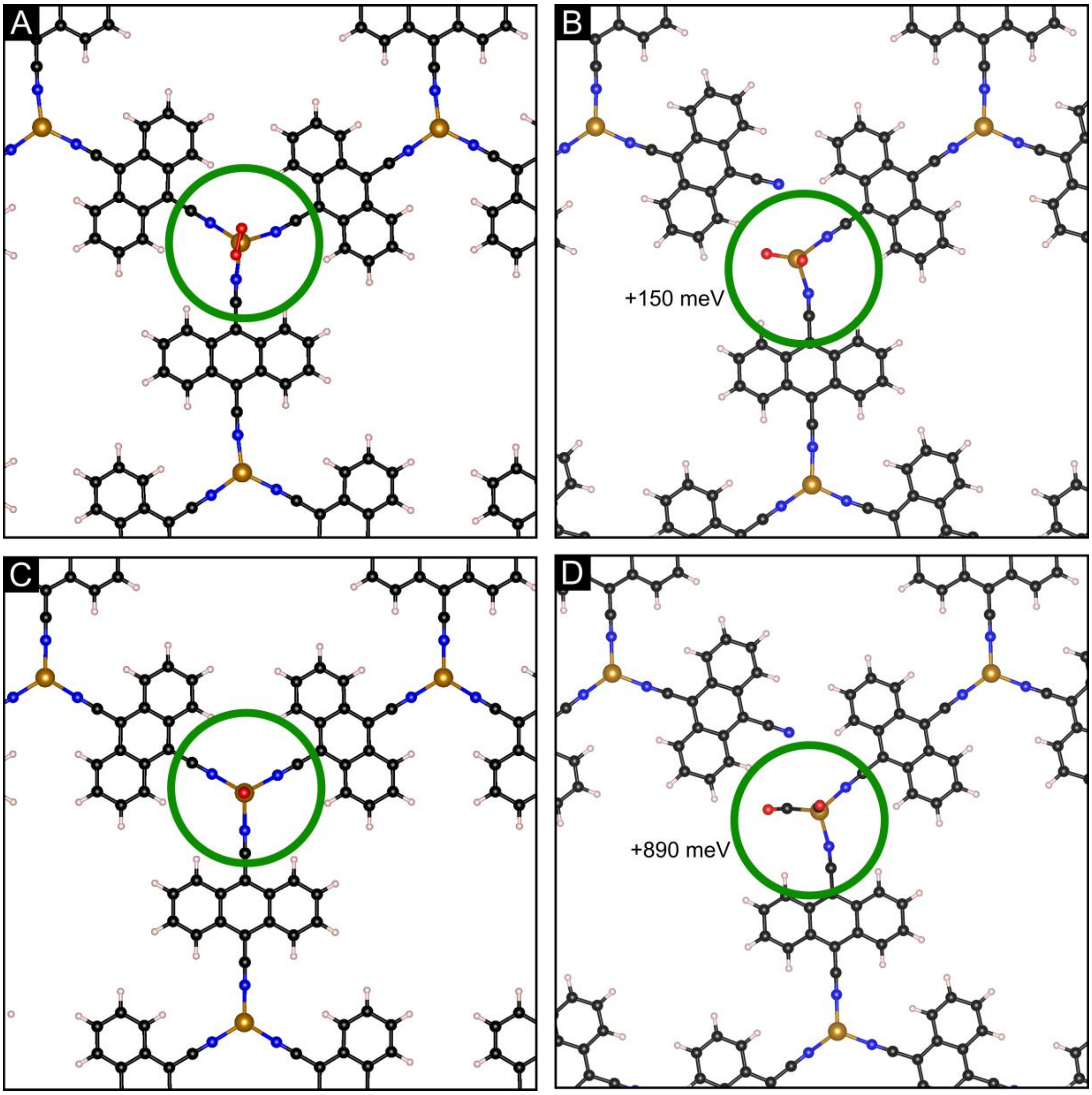

Figure S8: Calculated plausible intermediates of the O_2- and CO-assisted decomposition processes. Panel A shows side-on molecular adsorption of O_2 on the Fe-DCA network. Dissociative O_2 adsorption accompanied by cleavage of one Fe–N bond, shown in panel B, is only 150 meV higher in energy. Panels C and D show similar structures for adsorption of one and two CO molecules. Here, the intermediate with two CO molecules adsorbed at a single Fe site is unstable under realistic conditions.
